# Metabolic effect of telmisartan and losartan in hypertensive patients with metabolic syndrome

**DOI:** 10.1186/1475-2840-4-6

**Published:** 2005-05-15

**Authors:** Cristiana Vitale, Giuseppe Mercuro, Carlotta Castiglioni, Alessandra Cornoldi, Arianna Tulli, Massimo Fini, Maurizio Volterrani, Giuseppe MC Rosano

**Affiliations:** 1 Department of Medical Sciences and Rehabilitation, IRCCS San Raffaele, Rome, Italy; 2 Department of Cardiology, University of Cagliari, Calgliari, Italy

**Keywords:** angiotensin II receptor blockers, telmisartan, losartan, hypertension, metabolic syndrome

## Abstract

**Background:**

Metabolic syndrome is a cluster of common cardiovascular risk factors that includes hypertension and insulin resistance. Hypertension and diabetes mellitus are frequent comorbidities and, like metabolic syndrome, increase the risk of cardiovascular events. Telmisartan, an antihypertensive agent with evidence of partial peroxisome proliferator-activated receptor activity-gamma (PPARγ) activity, may improve insulin sensitivity and lipid profile in patients with metabolic syndrome.

**Methods:**

In a double-blind, parallel-group, randomized study, patients with World Health Organization criteria for metabolic syndrome received once-daily doses of telmisartan (80 mg, n = 20) or losartan (50 mg, n = 20) for 3 months. At baseline and end of treatment, fasting and postprandial plasma glucose, insulin sensitivity, glycosylated haemoglobin (HBA_1c_) and 24-hour mean systolic and diastolic blood pressures were determined.

**Results:**

Telmisartan, but not losartan, significantly (p < 0.05) reduced free plasma glucose, free plasma insulin, homeostasis model assessment of insulin resistance and HbA_ic_. Following treatment, plasma glucose and insulin were reduced during the oral glucose tolerance test by telmisartan, but not by losartan. Telmisartan also significantly reduced 24-hour mean systolic blood pressure (p < 0.05) and diastolic blood pressure (p < 0.05) compared with losartan.

**Conclusion:**

As well as providing superior 24-hour blood pressure control, telmisartan, unlike losartan, displayed insulin-sensitizing activity, which may be explained by its partial PPARγ activity.

## Background

Metabolic syndrome describes the presence of a cluster of common cardiovascular risk factors, including hypertension, insulin resistance or glucose intolerance, visceral obesity, atherogenic dyslipidemia, prothrombotic state and proinflammatory state in a single individual [1,2]. The lack of a universally agreed definition has impeded epidemiologic work on the prevalence and antecedents of this syndrome. Nevertheless, it has been proposed that the metabolic syndrome is present in about 10–25% of individuals in industrialized countries [3,4]. The increasing availability and abundance of high-calorie, low-fiber foods and the adoption of more sedentary lifestyles are also leading to increased prevalence of the metabolic syndrome in developing countries [5]. Its presence predicts a two- to four-fold increase in the risk of cardiovascular disease and death [6,7] and the risk of developing type 2 diabetes is increased five- to nine-fold [3,8].

In general, components of the metabolic syndrome are treated individually, there being no current treatment that targets all features. Some classes of antihypertensives, notably calcium channel blockers, angiotensin-converting enzyme (ACE) inhibitors and angiotensin II receptor blockers (ARBs), have been shown to reduce the incidence of new-onset diabetes, particularly when compared with diuretics and β-blockers [9]. This suggests that antihypertensive agents have differential effects on hyperglycemia in patients with metabolic syndrome. However, there are few data on intra-class differences. Recent in vitro and animal studies suggest that telmisartan, unlike other ARBs, acts as a partial peroxisome proliferator-activated receptor-gamma (PPARγ) agonist at concentrations that are achievable with oral doses recommended for the treatment of hypertension, thus suggesting its insulin-sensitizing effect [10-12]

The aim of the present study was to compare the glucometabolic effect of telmisartan and losartan, two ARBs with potentially different effects on glycemia, in patients with metabolic syndrome.

## Materials and methods

The study population included men and women aged between 18 and 75 years with arterial hypertension and the diagnosis of metabolic syndrome. All subjects were newly diagnosed as being hypertensive (office systolic blood pressure [SBP] ≥ 135 mmHg, diastolic blood pressure [DBP] ≥ 85 mmHg). Patients were required to have insulin resistance, impaired glucose tolerance (IGT) or type 2 diabetes, according to the diagnostic criteria for the metabolic syndrome of the World Health Organization [1]. Insulin resistance was defined as HOMA-IR > 3.5, impaired glucose tolerance (IGT) was defined as 2-hour values in the oral glucose tolerance test (OGTT) of ≥ 140 mg/dl (≥ 7.8 mmol/l), but <200 mg/dl (<11.1 mmol/l). Diabetes was diagnosed as free plasma glucose (FPG) ≥ 126 mg/dl (≥ 7.0 mmol/l) or 2-hour post-glucose load of ≥ 200 mg/dl (≥ 11.1 mmol/l). Patients with hyperkalemia or serum creatinine >2 mg/dl were excluded.

After evaluation of all inclusion and exclusion criteria, eligible patients entered a randomized, parallel-group, double-blind study. After a baseline 24-hour ambulatory blood pressure monitoring and an OGTT, they were assigned to the two treatment groups using equal weighting and electronic randomization, and received either once-daily telmisartan 80 mg or losartan 50 mg for 3 months. These dosages were employed because they are the highest approved for the treatment of hypertension on the basis of Italian licensing. Patients were asked to adhere to their standard eating habits and physical activity throughout the study.

Patients were assessed at baseline (first visit) and after 3 months' treatment. Fasting (minimum 12 hours) blood samples (10 ml) were obtained for laboratory evaluation of hematology and clinical chemistry parameters, including total cholesterol, LDL cholesterol, high-density lipoprotein (HDL) cholesterol, triglycerides, glucose and insulin. An OGTT was conducted using 75 g glucose. Blood samples (10 ml) were withdrawn at 30-minute intervals over 120 minutes for determination of glucose and insulin response. An autoanalyzer (Olympus) was used to assay plasma glucose using the hexokinase method, plasma triglycerides using the glycerol-3-phosphate oxidase-*p-*aminophenazone method; cholesterol using cholesterol oxidase phenol ampyrone method; HDL cholesterol using immunoinhibition; glycosylated hemoglobin (HbA_1c_) with the Abbott AxSYM analyzer (Abbott SpA Divisione Diagnostici, Rome, Italy) using microparticle enzyme immunoassay; and free plasma insulin (FPI).

Insulin resistance was measured using the homeostasis model assessment (HOMA-IR) [13], defined by the following formula:



Trough clinical blood pressures were recorded at baseline and after treatment using cuff sphygmomanometry. Ambulatory blood pressure monitoring (ABPM) was performed with an oscillometric device (Tonoport V; GE Medical Systems IT Inc., Milwakee, WI, USA) recommended for clinical use and that had previously been validated. The monitor was programmed to measure SBP and DBP every 20 minutes between 06.00 and 22.59 (daytime period) and every 30 minutes between 23.00 and 05.59 (night-time period). A standard, adult-sized arm cuff (length 12 cm) was positioned in the middle of the non-dominant arm covering the brachial artery above the antecubital fossa. The correct position for the cuff was confirmed when three SBP and DBP values were concordant (within 5 mmHg) with those obtained from the opposite arm with a standard sphygmomanometer. The arm cuff was inflated automatically by a pump, and the blood pressure was digitally recorded on the hard disk of a personal computer to which the device was connected. Patients were instructed to continue their usual daily activities, but to keep their arm still and parallel to the trunk during ambulatory blood pressure measurements, and to return to the hospital 24 hours after initiation of the ABPM.

ABPM data were excluded from analysis if >30% of the measurements were lacking, if data were missing for >3-hour spans, or if collected from patients who were experiencing an irregular rest-activity schedule or a night-time sleep span was <6 hours or >12 hours during ABPM. Mean SBP and DBP values for the daytime (06.00–22.59) and night-time (23.00-05.59) periods were calculated as mean values of the hourly averages. Smoothness index was calculated as the ratio of the standard deviation of the hourly blood pressure value to the 24-hour mean [14].

Body mass index (BMI) was measured as the ratio of weight (kg) to height (m^2^). Waist circumference was measured with a tape measure placed horizontally around the abdomen at the level of iliac ridge at the end of a normal expiration, keeping the tape well tense, adhered to the skin and parallel to the floor. Any adverse event was recorded.

Data are presented as mean ± 1 SD or percentages when appropriate. After testing data for normality, Wilcoxon Signed Rank test was used to compare values before and after each therapy and the relative changes in values in response to each therapy. The effects of the losartan and telmisartan on blood pressure and glucose metabolism were analyzed by one way repeated measures analysis of variance (ANOVA) or Friedman Repeated ANOVA on Ranks. A p value <0.05 was considered statistically significant.

## Results

A total of 40 patients were enrolled, with 20 randomized to each treatment group, baseline clinical characteristics of study patients are shown in table 1, no significant differences were noted between groups. All but four patients had IGT, whereas one patient in the losartan group and three in the telmisartan group had a diagnosis of type 2 diabetes.

**Table 1:** Patient baseline characteristics

	**Losartan (n = 20)**	**Telmisartan (n = 20)**	**P value**
Mean ± SD age (years)	56.2 ± 11.0	55.3 ± 12.4	NS
Males/females	11/9	12/8	
Office blood pressure (mmHg)			
Mean ± SD SBP	149.7 ± 9.0	151.3 ± 7.1	
Mean ± SD DBP	91.2 ± 7.4	89.8 ± 8.7	
24-hour mean blood pressure (mmHg)			
Mean ± SD SBP	142.8 ± 12.0	143.6 ± 14.0	NS
Mean ± SD DBP	88.8 ± 10.2	88.3 ± 9.5	NS
Mean ± SD body mass index (kg/m^2^)	32.1 ± 7.2	34.5 ± 6.3	NS
Impaired glucose tolerance (n)	19	17	NS
Type 2 diabetics (n)	1	3	NS
Metabolic syndrome components (n)^a^			
3	11	10	NS
4	7	8	NS
5	2	2	NS
Total cholesterol (mg/dl)	212.6 ± 45.8	209.6 ± 50.8	NS
Low-density lipoprotein cholesterol (mg/dl)	134 ± 44	138 ± 34	NS
High-density lipoprotein cholesterol (mg/dl)	51.2 ± 11	56.3 ± 17	NS
Triglycerides (mg/dl)	221 ± 32	210 ± 23	NS

^a ^Based on World Health Organization criteria [1].

**Figure 1:**
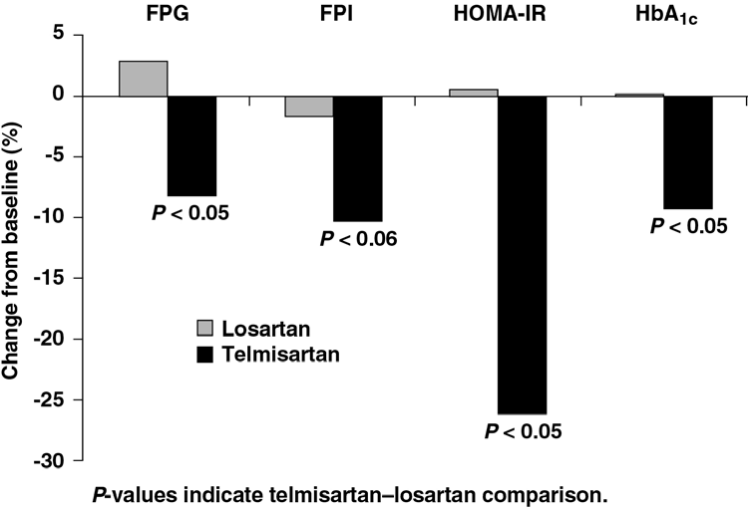
Effect of telmisartan and losartan on measures of glycaemia and insulin resistance in 40 patients with metabolic syndrome. FPG = fasting plasma glucose, FPI = fasting plasma insulin, HOMA-IR = homeostatic model assessment – insulin resistance, HbA_1c _= glycosylated haemoglobin.

**Figure 2:**
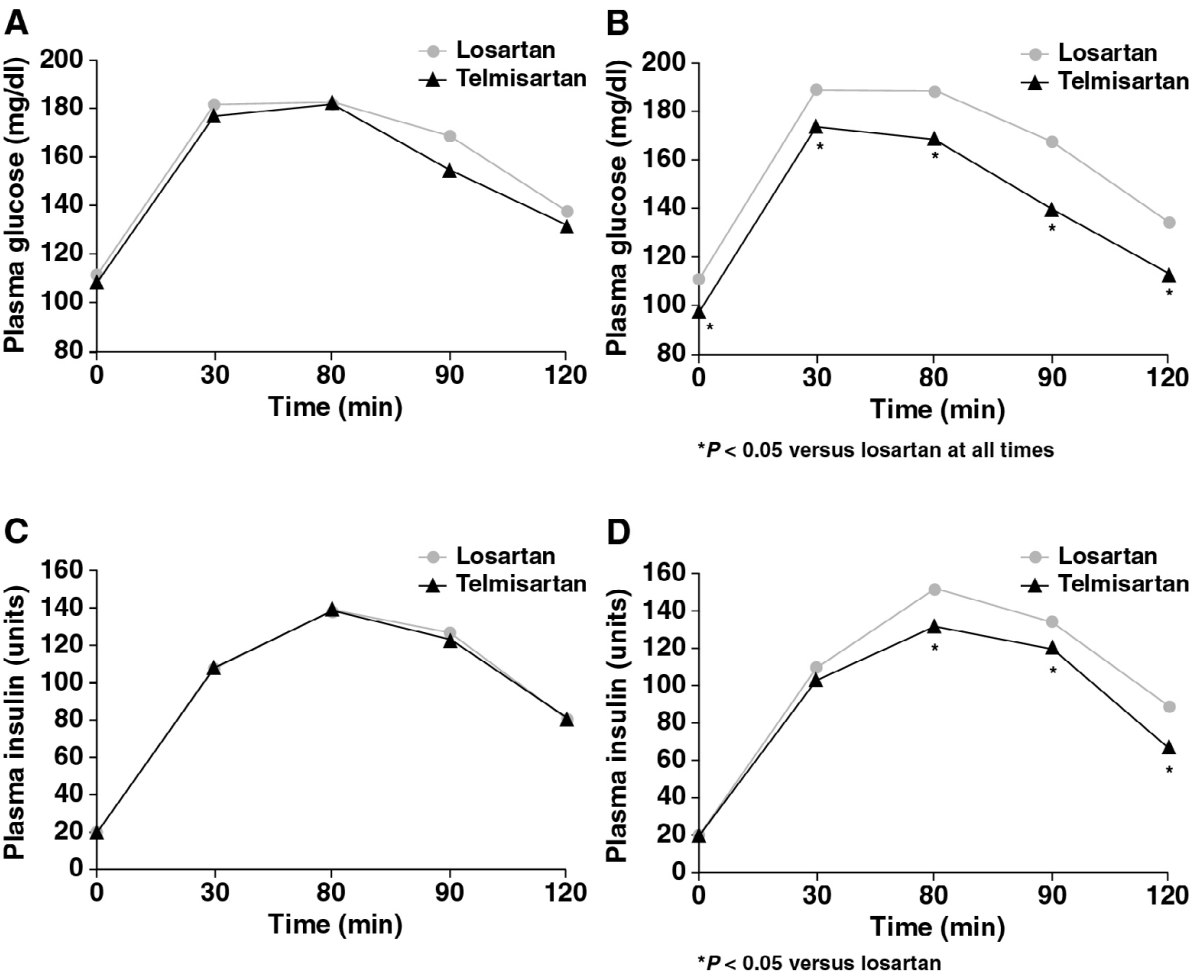
Effect of telmisartan and losartan during the OGTT in patients with metabolic syndrome. A) Plasma glucose at baseline. B) Plasma glucose at endpoint. C) Plasma insulin at baseline. D) Plasma insulin at endpoint.

**Figure 3:**
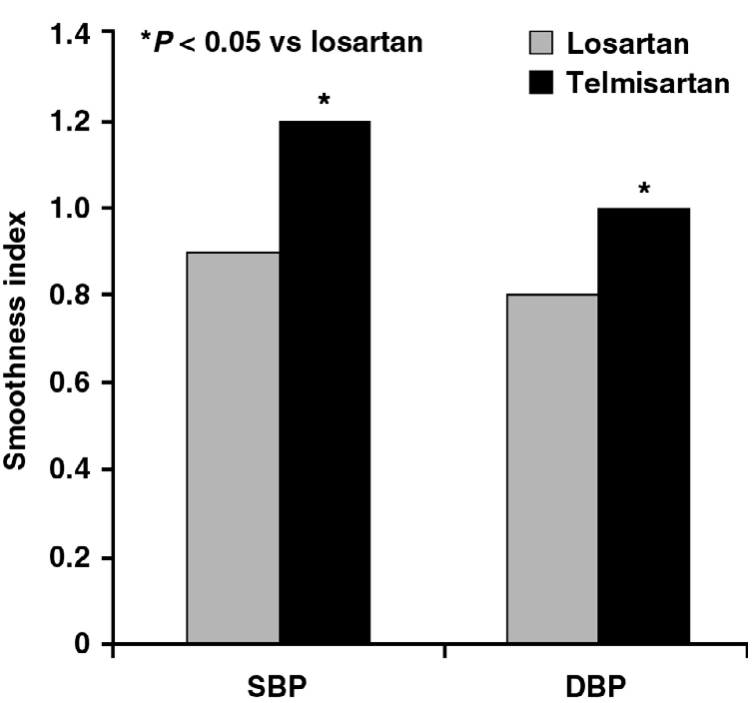
Effect of telmisartan and losartan on the smoothness index at endpoint.

Changes in metabolic parameters were observed at the end of treatment compared with baseline (table 2). Compared with losartan, telmisartan reduced FPG by 8% (p < 0.05), FPI by 10% (p < 0.06), HOMA-IR by 26% (p < 0.05) and HbA_1c _by 9% (p < 0.05) as shown in figure 1. Losartan did not have a meaningful effect on these parameters. Levels of FPG and FPI following OGTT were also significantly reduced by telmisartan compared with losartan (figures 2 and 3).

**Table 2:** Mean ± SD metabolic parameters at baseline and end of treatment

	**Baseline**		**p value**	**End of treatment**		**p value**
		
	**Losartan**	**Telmisartan**		**Losartan**	**Telmisartan**	
HOMA-IR	5.78 ± 3.53	5.74 ± 3.35	NS	5.82 ± 2.66	4.24 ± 2.36	< 0.05
FPG	110.05 ± 14.56	109.08 ± 16.67	NS	113.20 ± 12.68	100.00 ± 11.99	< 0.05
FPI	20.47 ± 9.64	18.86 ± 10.89	NS	20.14 ± 9.49	16.93 ± 8.91	< 0.06
2-hour OGTT	137.42 ± 32.5	131.31 ± 42.05	NS	134.6 ± 26.71	113.85 ± 42.14	< 0.01
HbA_1c_	6.27 ± 0.29	6.45 ± 0.35	NS	6.28 ± 0.21	5.85 ± 0.18	< 0.05

After 3 months' treatment, telmisartan reduced 24-hour mean SBP and DBP significantly more than losartan. The superior blood pressure control with telmisartan was also apparent when changes in mean daytime SBP (13.5 ± 0.8 vs 10.0 ± 1.1 mmHg; p < 0.05) and DBP (8.9 ± 0.6 vs 5.6 ± 0.8 mmHg; p = 0.04) and mean night-time SBP (8.7 ± 0.9 vs 5.6 ± 1.3 mmHg; p < 0.05) and DBP (7.8 ± 1.1 vs 4.7 ± 0.8 mmHg; p < 0.05) were compared. There was no significant correlation between the decrease in blood pressure and the change in FPG (r = 0.28; p = 0.020) or FPI (r = 0.036; p = 0.012). Telmisartan also improved the SBP and DBP smoothness indices (figure 3).

Both telmisartan and losartan were well tolerated, with no adverse events reported.

## Discussion

This study found that, compared with once-daily losartan 50 mg, once-daily telmisartan 80 mg reduced 24-hour mean blood pressure and blood pressure variability, and improved glucose tolerance and insulin sensitivity. Improvements were found in all three indices of glucose and insulin metabolism- FPG, OGTT and HbA_1c _suggesting that Telmisartan may be effective in reducing the progression of metabolic syndrome.

Losartan is an ARB that has been shown in the Losartan Intervention For Endpoint reduction in hypertension (LIFE) to reduce new-onset diabetes compared with atenolol [15]. However, β-blocker therapy is a risk factor for the development of diabetes [16,17], so the hyperglycemic effect of atenolol may explain this result. Telmisartan is an ARB with a longer duration of action than losartan [18]. Given once daily, telmisartan 80 mg significantly reduced 24-hour blood pressure compared with losartan 50 mg, with especially large benefit in the last 6 hours of the dosing interval [18].

There is also clinical evidence that telmisartan has favourable metabolic effects. Previous studies showed that telmisartan 80 mg, but not valsartan 160 mg has an insulin-sensitizing effect [19]. A 1-year study in patients with type 2 diabetes treated with telmisartan or eprosartan found that only telmisartan improved plasma lipid profiles [20], but did not significantly affect glycemia or insulin sensitivity. However, a relatively low dose of telmisartan (40 mg once daily) was used in that study and, since telmisartan acts as a partial PPARγ agonist, this may have been insufficient for a full manifestation of any hypoglycemic effects. Telmisartan has been shown to improve plasma total cholesterol and low-density lipoprotein (LDL) cholesterol compared with nifedipine gastrointestinal therapeutic system in patients with type 2 diabetes and mild hypertension [21]. Furthermore, in a German surveillance study of hypertensive patients receiving telmisartan, the patients with type 2 diabetes had substantially reduced plasma glucose and serum triglyceride concentrations after 6 months' treatment [22].

FPG is the standard test used to diagnose Type 2 diabetes, but it is also a marker for cardiovascular disease in its own right [23]. Non-diabetic individuals with an FPG ≥ 100 mg/dl (≥ 5.6 mmol/l) but <126 mg/dl (<7.0 mmol/l) are considered to have impaired fasting glucose and are at increased risk of cardiovascular complications [24]. The physiologic basis of the response to OGTT differs from that of impaired FPG, with postprandial hyperglycemia closely linked to a blunting of early-phase insulin release [25]. It is often one of the earliest abnormalities that can be detected in clinical practice (although OGTT is not recommended for routine clinical use). HbA_1c _provides an index of plasma glucose concentrations during the previous 2–3 months [26]. It reflects both fasting and postprandial plasma glucose and, therefore, represents an independent parameter [27]. In patients such as ours, with high-normal levels of HbA_1c_, it is more closely related to postprandial plasma glucose than to fasting values [27,28].

In addition to these measures of glycemia, this study also used the HOMA-IR index, a measure of insulin resistance derived from fasting levels of glucose and insulin and a physiologically-based model [29], which is an effective, easily-derived surrogate for the more complex euglycemic clamp [30]. HOMA-IR was predictive of future diabetes in the Mexico City Diabetes Study [31]. The reduction of HOMA-IR seen in our study may, therefore, reduce the progression from metabolic syndrome, although this has not been studied experimentally.

In this study, losartan had no effect on measures of glycemia or on HOMA-IR; a result that may seem surprising given that losartan reduced the incidence of new-onset diabetes in LIFE [15]. However, a previous study in hyperinsulinemic, hypertensive patients found no effect on insulin sensitivity or glucose tolerance following 12 weeks' treatment with either losartan or metoprolol [32]. This supports our finding that losartan is metabolically neutral, but leaves open the question of whether the results of LIFE were due to a pro-diabetogenic effect of atenolol. They also support previous studies which suggest that the PPARγ agonism exhibited by telmisartan in preclinical studies [10-12] has meaningful effects at the clinical level.

There have been relatively few studies of PPARγ agonists in patients without diabetes. In one study, 24 hypertensive, non-diabetic patients (mean BMI of 30 kg/m^2^) were given rosiglitazone in addition to non-ACE inhibitor antihypertensive therapy for 12 weeks. The result was a reduction in FPI (but not FPG) and an increase in glucose disposal (measured using euglycemic clamp) [33]. In an 8-week, placebo-controlled study, 50 non-diabetic patients who met a modified National Cholesterol Education Program definition for the metabolic syndrome were randomized to receive either rosiglitazone 4 mg/day or placebo for 8 weeks. In these patients, rosiglitazone reduced FPI by 40% and HOMA-IR by 45% compared with placebo [34]. The magnitude of the sensitizing effect with rosiglitazone was somewhat greater than that observed with telmisartan in our study. Although these difference may be a function of the differing population and study protocol, they may also relate to the in vitro observations that telmisartan is a selective PPARγ modulator (SPPARM) [10]. SPPARMs activate only a subset of genes targeted by full PPARγ agonists [35] and they may, in particular, have a better adverse event profile. For this reason, it is notable that telmisartan was well tolerated in our study as in previous ones, with none of the peripheral oedema and fluid retention that are characteristic of full PPARγ agonists [36].

As expected, both telmisartan and losartan reduced blood pressure in our patients; however, reductions in 24-hour mean SBP and DBP were significantly greater with telmisartan. A superior reduction in 24-hour mean SBP and DBP with telmisartan 80 mg compared with losartan 50 mg has been found in a meta-analysis of previous studies, partly due to telmisartan's longer duration of action [18]. The greater improvement in the smoothness index with telmisartan compared with losartan is also significant, given that this is an independent prognostic marker for cardiovascular events [37].

Compared to other AT(1) receptor blockers telmisartan may have further additional beneficial effects in patients with the metabolic syndrome as suggested by this study and by a recent report of Zhang et al that have shown that that AT(1) receptor-mediated coronary constriction that is augmented in the prediabetic metabolic syndrome and contributes to impaired control of coronary blood flow is beneficially affected by telmisartan[38].

## Conclusion

This study found that telmisartan, but not losartan, improves metabolic parameters in patients with metabolic syndrome. Although treatment conventionally focuses on each risk factor individually, multifactorial intervention reduces significantly the incidence of cardiovascular disease in type 2 diabetics with microalbuminuria. The multifactorial effects of telmisartan shown in this study could, therefore, provide synergistic benefits in patients with hypertension and other cardiovascular risk factors, such as glucose intolerance. Such a provocative hypothesis will require confirmation in large clinical trials, such as the ONgoing Telmisartan Alone and in combination with Ramipril Global Endpoint Trial (ONTARGET) [39].

## Competing interests

The author(s) declare that they have no competing interests.

## Authors' contributions

All authors contributed to the conduct of the study. GR conceived the study and prepared the manuscript.
